# New Au/chitosan nanocomposite modified carbon paste sensor for voltammetric detection of nicotine

**DOI:** 10.1038/s41598-023-47703-7

**Published:** 2023-11-22

**Authors:** M. Shehata, M. Zaki, Amany M. Fekry

**Affiliations:** Chemistry Department, Faculty of Science, Cairo University, Giza, 12613 Egypt

**Keywords:** Chemical biology, Chemistry

## Abstract

A profoundly touchy voltammetric sensor for detection of nicotine (NIC) in urine and tobacco specimens has been developed in light of the boosted electrochemical response of NIC at gold and chitosan nanocomposite modified carbon paste electrode (ACMCPE). Material characterization techniques Scanning Electron Microscope and Energy Dispersive X-ray (SEM & EDX) were utilized to describe the ACMCPE surface material. The impedance spectroscopy technique (EIS), cyclic voltammetry (CV), chronoamperometry (CA), and differential pulse voltammetry (DPV) were employed to explore the electrochemical sensing of NIC at ACMCPE. The created sensor exhibits an exceptional electrochemical sensitivity to NIC in a universal Britton–Robinson (B-R) buffer solution with a pH range of 2.0 to 8.0. The sensor shows a linear response over NIC concentration ranges of 4.0–320.0 µM, with the detection limit (LOD) of 7.6 µM. The prepared sensor has been shown to be exceptionally viable in detecting NIC with amazing selectivity and reproducibility. We suggest it as a trustworthy and useful electrochemical sensor for NIC location.

## Introduction

NIC is perhaps of the most well-known normal alkaloid, and it's the primary component in tobacco plants that is generally used to produce cigarettes^1,2^. When consumed through smoking, this substance might cause different adverse consequences on human health, involving elevated blood pressure and heart rate, as well as central nervous system excitement^3–7^. Thus, from a toxicological and medical standpoint, checking NIC levels is mandatory. Numerous techniques have been utilized to detect NIC in the human body or tobacco specimens, including spectrophotometry^8^, HPLC technique^9^, gas chromatography^10^ and capillary electrophoresis^11^. Some of these approaches, however, have shortcomings, such as the need of sample pretreatment, expensive prices, and long processing time. Electroanalytical strategies, which can give high awareness, fast response, simplicity of activity, and minimal expense, are ideal options in this regard^12–14^. Nanomaterials have been generally utilized in sensors and biosensors because of their novel properties, for example, the presence of conduction centers, facilitating electron transfer and providing large catalytic surface areas, as well as enhanced diffusion and adsorption towards target molecules. In recent years, various nanomaterials for electrode surface modification have been reported^15–18^. One of the new encouraging materials, Au nanoparticles (Au-NPs), were generally used to improve electrodes. This choice was made essentially as a result of Au-NPs have consistent physical and chemical resources, useful catalytic activities, and little dimensional size^19,20^. Based on these advantages, it has been proved that, modifying carbon paste electrodes (CPEs) with Au-NPs improves sensitivity of the electrode, facilitates charge transfer, and lowers the analyte detection limit. For example, Afkhami et al.^21^ developed a sensor that detects cefixime in urine and pharmaceutical samples using a multi walled CPE (Au-NPs/MWCPE) modified with Au-NPs. The benefits of this sensor are its extremely low detection threshold, elevated sensitivity, and outstanding stability. Arvand et al.^22^ developed a sensor for measuring folic acid in human blood plasma using an Au-NPs-modified CPE. This sensor has a fast response time and a high sensitivity. Recently, a few investigations on the detection of NIC utilizing Au-NPs nanoparticles have been conducted. For instance, Saljooqi et al.^23^ reported electrocatalytic action of the composite GCE/GO, and polythiophene (PT) decorated with Au-NPs enabling anodic peak current of NIC oxidation to be noticed at the potential of + 0.81 V and limit of detection 1.7 × 10^–7^ mol mL^−1^. Whilst, Jing et al.^24^ have revealed a detection limit of 0.015 μM at PDA-RGO/Au nanocomposite sensor in tobacco products. More recently, the synthesis of nanocomposite of Au-NPs with chitosan has received a lot of interest. Chitosan, a deacetylated derivative of chitin, is rich in amino groups as being a linear polysaccharide, exhibits good biocompatibility, owing prominent electronic properties and has an excellent stability which allows good electrocatalytic activity, large specific surface area and high sensitivity towards NIC when combined with the well biocompatible Au-NPs. The strong interest in this polymer stems from its appealing features, which include outstanding high-water permeability, strong adhesion, and film-forming properties, and chemical modification susceptibility as a result of the existence of reactive amino and hydroxyl functional groups^25–29^. These functional groups could contribute to the settlement of Au-NPs on the surface of the electrode as well as the electrode surface's catalytic activity. Zhang et al.^30^ constructed a sensor based on chitosan and Au-NPs. They found that chitosan averted the agglomeration of the Au-NPs as well as maintained the stability of nanoparticles. For the creation of electrochemical sensors, it is in fact especially appealing to create hybrid materials that combine highly conductive Au-NPs with a wide range of organic functional groups capable of selectively interacting with target molecules. In earlier works, two highly sensitive NIC detection sensors using CPE modified with Ni/Cu NPs and Mn/Cu NPs have been constructed by M. Zaki et al.^31,32^. In this work, a novel NIC sensor with outstanding detection properties was created by electrodepositing Au -NPs onto a chitosan/CPE surface.

## Experimental

### Reagents and solutions

The standard NIC analyte sample (99%) was supplied by the Egyptian Smoking Eastern Company and was used without prior refinement. The freshly made 1.62 g L^−1^ NIC stock solutions in water were then stored in a dark container due to the compound's light sensitivity. Paraffin oil mixed with graphite fine particles, supplied by Merck, were used to set up the CPE. Gold chloride salt (KAuCl_4_) and chitosan powder were provided by Aldrich (USA). (B–R) buffer solutions (4.0 × 10^–2^ M), utilized as supporting electrolytes, were prepared from CH_3_COOH, H_3_BO_3_ and H_3_PO_4_ at pH values ranging from 2.0–8.0. PH values were changed utilizing HCl (0.2 M) and NaOH (0.2 M). Each experiment produced repeatable findings after being run two to three times at laboratory room temperature.

### Modified electrodes construction

A homogenous paste was made by mixing 2 mL of paraffin oil with 5.0 g of graphite powder in a mortar for 5 min to prepare the bare carbon paste electrode (denoted as BCPE)^33,34^. In order to find the optimal sensor composition, several attempts were made to create the modified CPE with chitosan by mixing various amounts of chitosan into the paste and assessing their response toward NIC. On that principle, 0.4 g of CP and 0.1 g of chitosan powder were prepared to create a Chitosan/CPE (denoted as CCPE). Every composite combination was then stuffed into the finish of a Teflon tube (a hole of 3 mm in diameter which equals 0.0706 cm^2^ electrode surface area) and polished with ultrafine emery paper to obtain a smooth surface. In order to prepare the Au-NPs/Chitosan/CP electrode (denoted as ACMCPE), the CMCPE was submerged in a solution of gold chloride (0.01 M) for 300 s to deposit Au-NPs on the surface of the CMCPE at a deposition potential of -0.4 V. It was then dried for 5 min by air.

### Cell and devices

The electrochemical investigations were carried out at room temperature using a three-electrode cell with a capacity of 25 mL, consisting of an ACMCPE as the working electrode, a platinum auxiliary electrode (CE), and a calomel reference electrode (RE). Electrochemistry techniques such as DPV, CV, CA and EIS were carried out by SP-150 potentiostat connected to the EC-Lab® software package**.** EIS measurements were carried out at 10 mV ac amplitude in the frequency range of 1.0 mHz to 100 kHz. EC-Lab^®^ programming was utilized to fabricate the best comparable circuit models^35–37^. pH measurements were performed using a pH-meter from Hanna Instruments in Italy. Energy Dispersive X-ray analyzer (Bruker) and SEM (TESCAN VEGA3, Czech Republic) were used to evaluate the electrode's surface morphology. Transmission electron microscopy (TEM) analysis was made using a JEM-1400 Electron Microscope (JEOL, Japan).

### Analysis of urine

Real urine samples were examined using a diluted pee solution created by diluting 400 times in 100 mL of B-R buffer pH 2 in order to create a stock solution. A solution containing NIC in B-R buffer at pH = 2 was used to establish standard increments of NIC at different concentrations to get the calibration graph.

### Cigarette sample examination

Cigarettes were dried for 30 min on a 40 °C stove after being taken out of their rolling papers. 10 mL of water and 0.1 g of tobacco from a combination of 10 cigarettes (from two packs of a comparable brand) were added, and the mixture was then sonicated for 3 h in an ultrasonic water bath and filtered. The B-R buffer (pH 2.0) was mixed with the appropriate volume (100 mL) of the filtrate and evaluated under the same conditions as those used to obtain the calibration graph^5^.

## Results and discussion

### Morphology of the investigated electrodes

**Figure 1:**
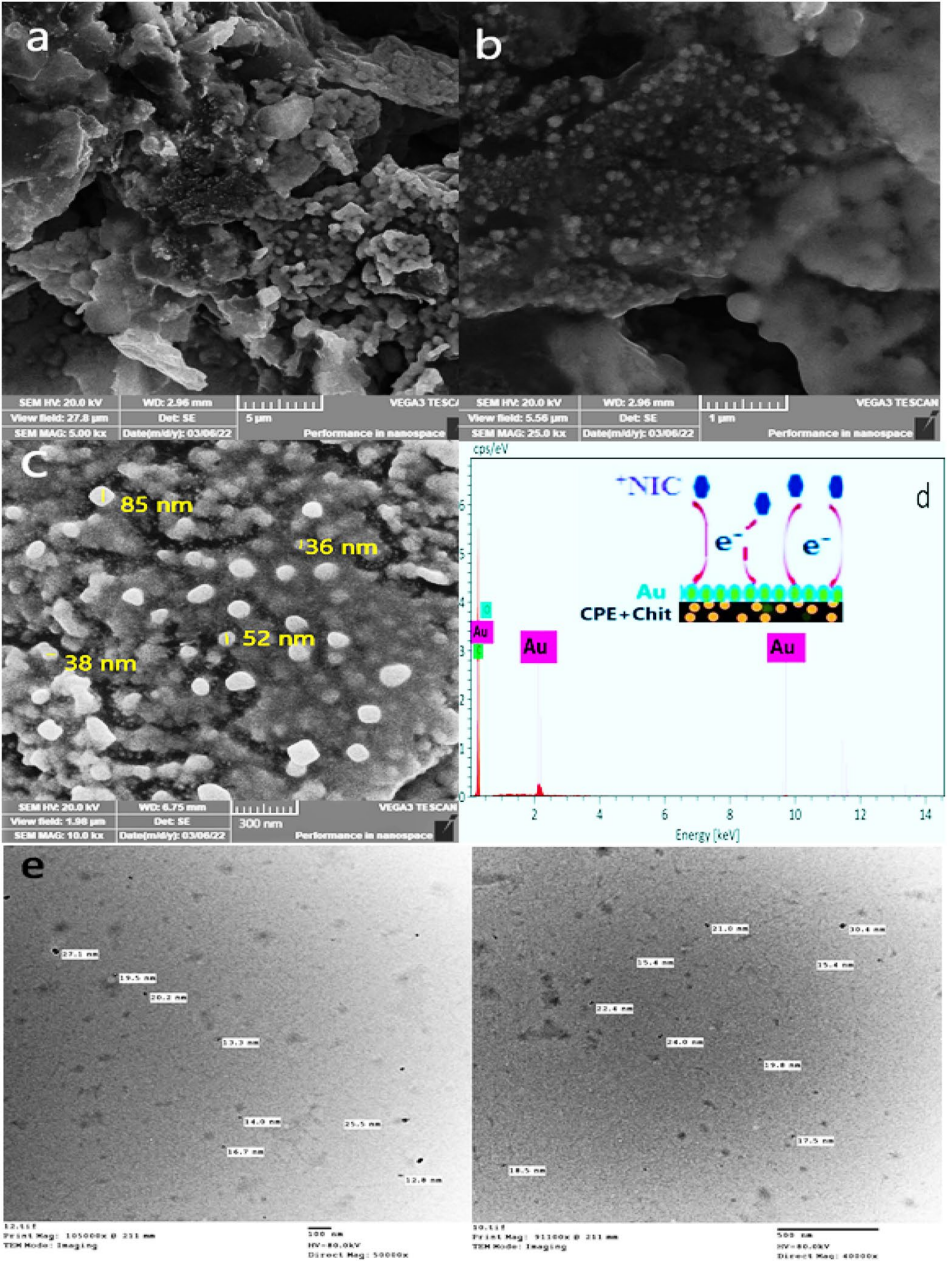
ACMCPE SEM pictures (**a**–**c**) at various amplifications. (**d**) EDX spectra of ACMCPE surface including NIC/ACMCPE interaction model. (**e**) Transmission electron microscopy (TEM) micrograph of Au nanoparticles.

SEM micrographs were used to examine the surface morphology of the Au-NPs that had been deposited on Chitosan/CPE. The Au-NPs have an approximately spherical shape and are dispersed randomly at varying altitudes over the electrode surface, as indicated by the SEM images of the ACMCPE surface at several amplifications shown in Fig. 1a–c. This suggests a significant surface area. According to the scale of the image, the size of Au-NPs varies between 30 and 50 nm as depicted in Fig. 1c. Only 85 nm particle was odd one and not repeatable across the whole prepared surface, which enhanced our surface stability and its well preparation procedure. Also TEM images enhanced our findings and the sizes were nearly from 15–30 nm as reported in Fig. 1e. The different particle sizes observed might be explained by nucleation, agglomeration, and subsequent growth. The EDX analysis of the ACMCPE sensor is shown in Fig. 1d, which verifies the presence of C, O, and Au by a percentage of 84.57%, 1.09%, and 14.34% respectively, confirming that Au-NPs were indeed coated on the Chitosan /CPE surface.

### Electrochemical measurements

**Figure 2:**
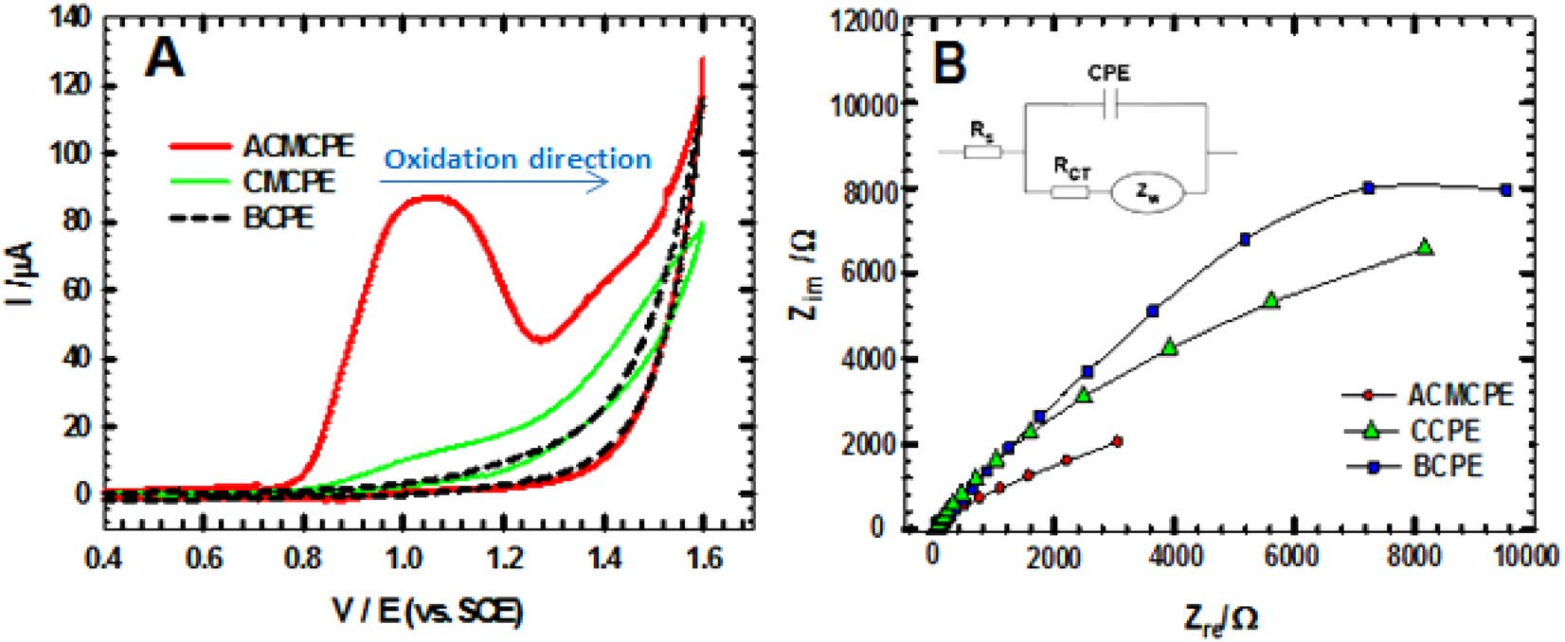
(**A**) CVs of BCPE, CCPE and ACMCPE at ν = 50 mV s^−1^in 1 mM NIC utilizing B-R buffer (pH 2). (**B**) Nyquist plots for BCPE, CMCPE and ACMCPE in 1 mM NIC using B-R buffer pH 2. Inset: equivalent circuit model.

The CV and EIS were used to evaluate the electrochemical properties of NIC at CPE, CCPE, and ACMCPE. Figure 2A shows typical voltammograms of 1.0 mM NIC in a B-R buffer (pH 2) produced by scanning the three working electrodes at a rate of 50 mV s^−1^. The oxidation of NIC at ACMCPE results in an irreversible oxidation peak (voltammogram ACMCPE) at + 1050 mV and Ip = 82.35 μA. In contrast, no redox response was observed for BCPE and a small Ip = 8.9 µA at a potential of around + 1000 mV was observed for CCPE, suggesting that the electrodeposition of Au-NPs on to the surface of the chitosan/CPE was a successful technique for improving NIC detection.

The Randles–Sevcik equation^38–40^ was utilized to determine the active surface area of the modified electrode for a known K_4_[Fe(CN)_6_] concentration:

1 $${\text{Ip}}\, = \,{2}.{69}\, \times \,{1}0^{{5}} \,{\text{n}}^{{{3}/{2}}} \,{\text{A D}}^{{{1}/{2} }} \,\nu^{{{1}/{2}}} \,{\text{C,}}$$

where Ip is the peak current (A), ν is the scan rate (V s^−1^), A is the electrode area, n is the number of transferred electrons, D the diffusion coefficient which equals to 7.6 × 10^–6^ cm^2^ s^−1^, and C is the concentration of K_4_[Fe(CN)_6_]. For 1.0 mmol L^−1^ K_4_[Fe(CN)_6_] in 0.10 mol L^−1^ KCl electrolyte with n = 1.

The bare electrode area was calculated using the hole diameter of 3 mm using πr^2^ = 0.0706 cm^2^ and after deposition of Au-NPs, the active surface area of ACMCPE was increased to 0.144 cm^2^.

Nyquist plots were exhibited at + 1050 mV (E_p_ from CV) for ACMCPE, CCPE, and BCPE (Fig. 2B). In the high-frequency region, the semicircle diameter of EIS corresponds to the charge transfer resistance (R_CT_) at the electrode electrode/electrolyte interface. A comparison of the semicircular regions of the three EIS curves at high frequency shows that the modified electrode ACMCPE has a lower R_CT_ than the bare electrode and CCPE^41–43^. The Randles circuit model was used to fit the acquired EIS curve (Fig. 2B inset) including R_CT_, solution resistance (R_s_), constant phase element of capacitance (CPE) and Warburg impedance (Z_w_)^44–46^. The fitting was done with EC-Lab^®^ software supplied with the SP-150 workstation. The ACMCPE electrode was shown to have higher values for Z_w_ (1380 Ω cm^2^ s^−1/2^), CPE (18.7 µF cm^−2^), and a lower value for the R_CT_ (2243 Ω cm^2^) demonstrating greater conductivity than bare CPE and CCPE with CPE = 6.3 µF cm^−2^, W = 456 Ω cm^2^ s^1/2^ and R_CT_ = 12,960 Ω cm^2^ and CPE = 8.6 µF cm^−2^, W = 488 Ω cm^2^ s^−1/2^ and R_CT_ = 11,250 Ω cm^2^ respectively. Accordingly, the considerable decrease in R_CT_ for ACMCPE compared to BCPE and CCPE can be attributed to Au-NPs deposited on CMCPE, which forms many electrocatalytic centers, lowering the contact resistance between ACMCPE/electrolyte interfaces. These findings back up the high anodic current I_p_ value obtained from CVs for ACMCPE electrodes.

### Influence of pH

**Figure 3:**
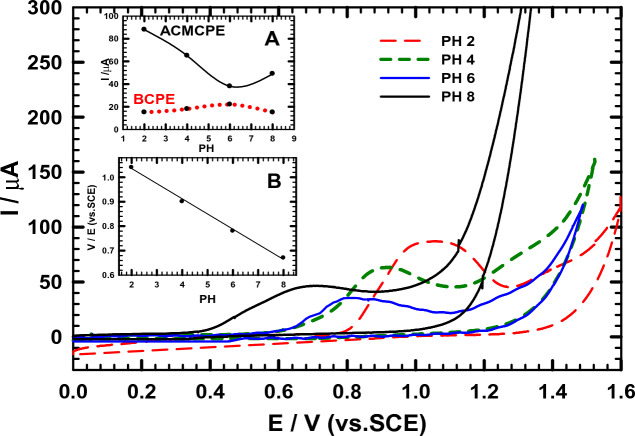
CVs of NIC at 50 mV s^−1^ using ACMCPE at different pH levels in B-R buffer. Inset: (**A**) A plot of pH vs. *I*_*pa*_ at BCPE and ACMCPE. (**B**) A plot of Ep vs. pH at ACMCPE.

The cyclic voltammogram approach was used to assess the impact of pH on NIC oxidation peak utilizing B-R buffer (pH 2–8) at both the BCPE and ACMCPE. According to Fig. 3, the anodic Ep moved negatively with rising pH values, demonstrating that protons are involved in electrode reactions and that the electrocatalytic oxidation of NIC was a pH-dependent process^47–49^. In addition, the linear relationship between Ep for NIC oxidation and pH (Ep = − 0.06 pH + 1.16 (R^2^ = 0.99) was demonstrated (see Fig. 3 inset B). According to the slope value, the electrode reactions demand an equal number of electrons and protons. At acidic and basic media, it is also obvious that peak potential varies with pH. At pH 2, the maximum peak current signal was obtained, which gradually decreased from 2.0 to 6.0 before increasing at pH 8. Accordingly, depending on the pH of the media, NIC has two pKa values: 3.12 and 8.02, which are equivalent to the monoprotonated (protonation of pyrrolidine nitrogen) and diprotonated (pyridine nitrogen) forms of the NIC molecule, respectively^50,51^. These results could indicate that NIC oxidation took place via protonation of nitrogen atoms on the pyrrolidine ring and was assigned to tertiary nitrogen oxidation.

**Figure 4:**
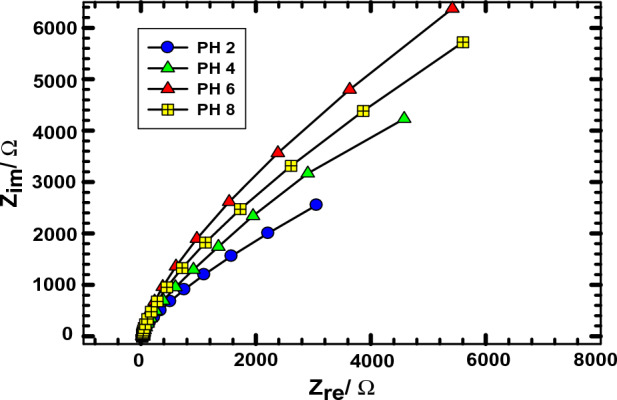
Nyquist plots of 1 mM NIC in various pH values utilizing ACMCPE.

Figure 4 shows Nyquist plots of NIC at different pH values utilizing ACMCPE at E_p_ vs. SCE. The EIS results are consistent with those obtained from CV. At different pH solutions, the Nyquist plot of ACMCPE shows almost straight lines at lower frequencies and tiny incomplete semicircles at higher frequencies, which correspond to diffusion-limited and electron transfer-limited processes^52–54^. The Nyquist plot at pH 2 shows a substantially smaller semicircle in the high-frequency region than other pH solutions, implying that ACMCPE at pH 2 has greater electron transport capabilities.

### The influence of scan rate

**Figure 5:**
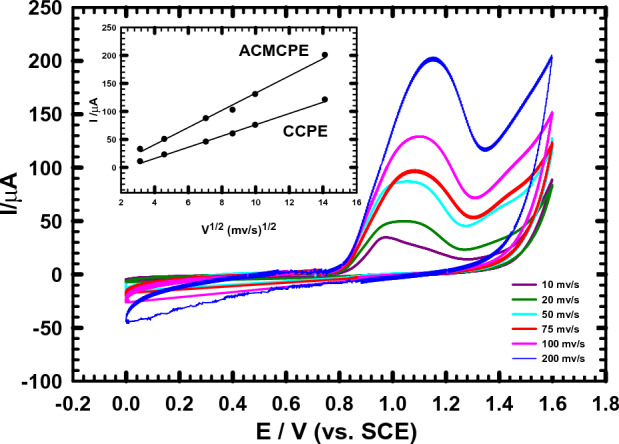
Influence of changing the scan rate ν from (10–200) mV s^−1^ on I_pa_ response of 1 mM NIC in B-R buffer (pH 2). Inset: Plot of the anodic peak currents Ip vs. ν^1/2^ for ACMCPE and CCPE.

In order to investigate the kind of the electrochemical process on the ACMCPE electrode, the impact of the scan rate (v) on the electrooxidation of NIC (1 × 10^–3^ M) was studied at various scan rates ranging from 10 to 200 mV s^-1^ using CV in B-R buffer (pH 2). It is clear that, in Fig. 5, as the scan rate increased, the peak potential (Ep) of the oxidation of NIC went considerably to higher positive values, demonstrating the irreversibility of the reaction^55,56^. A plot of the anodic peak currents (Ip) and the square root of scan rate (v^1/2^) revealed good linearity (Ip = 15.2 × ν^1/2^ + 20.6) with a correlation coefficient of r^2^ = 0.988 (see Fig. 4 inset), indicating that the charge transfer was a diffusion-controlled process^57,58^. In addition, the diffusion coefficients, D_app,_ of NIC in B–R buffer (pH 2.0) utilizing ACMCPE and CMCPE were derived from the relationship between the squire root of the scan rate v^1/2^ and the anodic peak current I_pc_ (A) [Fig. 5 inset] based on the Randles–Sevcik equation^59–61^.

2 $${\text{I}}_{{\text{p}}} \, = \,0.{4463}\,{\text{nFAC}}_{0} \,\left( {{\text{nFvD}}/{\text{RT}}} \right)^{1/2} .$$

In this equation, n is the number of electrons exchanged in oxidation at T = 298 K, C_0_ is the analyte concentration (1 × 10^−6^ mol cm^−3^), D is the electroactive species diffusion coefficient (cm^2^ s^−1^), F is the Faraday constant in C mol^−1^, ν is the scan rate in V s^−1^, A is the geometrical electrode area (cm^2^), and R is the gas constant in VC K^−1^ mol^−1^. Thus, the apparent diffusion coefficients, D_app,_ of NIC at ACMCPE and CMCPE were found to be 8.841 × 10^−5^cm^2^ s^−1^ and 5.12 × 10^−5^cm^2^ s^−1^, respectively.

### Chronoamperometry

**Figure 6:**
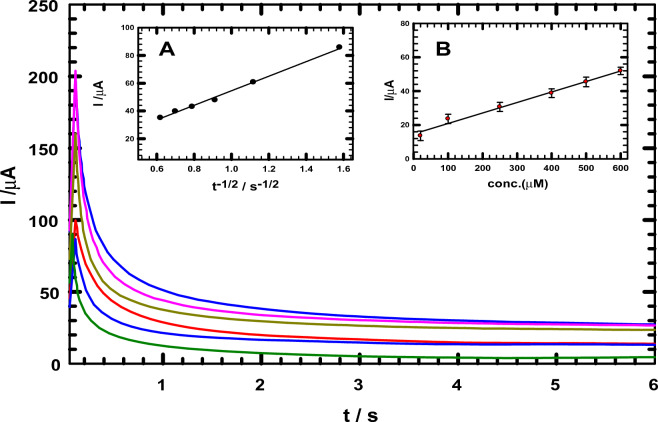
Chronoamperograms obtained at ACMCPE with varying doses of NIC (20, 100, 250,400, 500, and 600 μM) in B-R buffer pH 2. Inset: (**A**) I vs. t^−1/2^ figure obtained from 600 µM NIC chronoamperogram. (**B**) Calibration plot for various NIC concentrations at a given period of 1 s. Error bars represent the standard deviations of three repetitive tests.

The chronoamperometric response of NIC in B-R buffer (pH 2.0) was recorded at a constant potential (+ 1050 mV vs. SCE) utilizing ACMCPE sensor. The NIC calibration graph, shown in Fig. 6 inset B, was created by plotting NIC concentrations versus oxidation currents at a given time (1 s). A linear relationship was observed from 20 to 600 μM with a linear equation of Ip = 0.062 C + 15.74, and a correlation coefficient of 0.96. Based on the Cottrell equation^62–64^, the diffusion coefficient can be calculated from the equation.

3 $$i = \left[ {\frac{{nFAc^{0} \sqrt D }}{{\sqrt {\pi t\;} }}} \right],$$

where i = current, in unit A, F = Faraday constant (96,485 C/mol), n = number of electrons, c^0^ = bulk concentration of the analyte in mol/cm^3^; A = area of the electrode in cm^2^, D = diffusion coefficient for species in cm^2^/s and t = time in s. The slope obtained from the relationship between the anodic peak current and t^-1/2^ (Fig. 6 inset A) equals nFAc^o^(D/π)^1/2^, in accordance with the Cottrell equation, so the diffusion coefficient of NIC was calculated to be 7.38 × 10^–5^ cm^2^ s^-1^. The NIC oxidation reaction is shown to be controlled by a diffusion control process, since the diffusion coefficient values estimated from chronoamperometric measurements (CA) are equivalent to those calculated from CV measurements.

### Calibration plot

**Figure 7:**
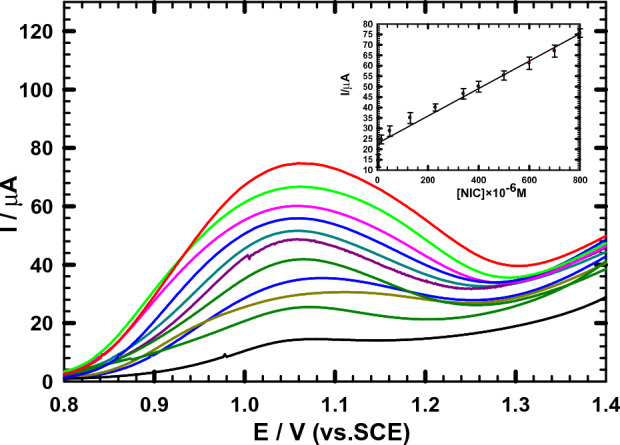
Effect of NIC addition in B-R buffer (pH 2.0) sequentially utilizing ACMCPE at 10 mV s^−1^. Inset: NIC calibration curve utilizing ACMCPE. The standard deviations of three repeated tests are shown as error bars.

The DPV response of ACMCPE electrode to different NIC concentrations in B-R Buffer (pH 2) was measured at 10 mV s^−1^, (Fig. 7). A distinct response was noted with the sequential addition of NIC, as indicated in Fig. 7. These results reveal that Au-NPs coated on a chitosan/CPE surface have a stable and efficient catalytic capability. Under optimal conditions, the response currents and NIC concentrations have a linear relationship in the range 4 × 10^–6^–3.2 × 10^–4^ M. The plot of anodic peak currents vs. NIC concentrations (see Fig. 7 inset) demonstrated a linear regression with the following equation: Ipa (A) = 0.065 C + 22.76 and r^2^ = 0.96. The following equations have been used to calculate the limits of detection (LOD) and quantification (LOQ):

4 $${\text{LOD }} = {\text{ 3s}}/{\text{m}}$$

5 $${\text{LOQ }} = { 1}0{\text{s}}/{\text{m}},$$

where m is the slope (μA/M) of the calibration curves and s is the standard deviation (SD) (three runs); they were found to be 7.6 × 10^−6^ M and 2.3 × 10^−6^ M, respectively^65,66^.

Comparisons of data acquired for determination of NIC using different instruments and electrochemical approaches versus ACMCPE were recorded in Table 1. Although it has roughly the same specific selectivity, the sensor has the benefit of employing non-poisonous and less expensive reagents. The ACMCPE sensor is less sophisticated and expensive than spectrometry equipment, HPLC, GC, and flow injection. Additionally, the pre-treatment and processes for these technologies are challenging, in contrast to how easy and uncomplicated they are for the ACMCPE sensor.

**Table 1:** Contrasting the recommended method with other techniques and electrodes for NIC measurement.

Method	Calibration range (M)	Detection limit (M)	Reference
Cho enzyme biosensor	9.2 × 10^−5^–2.0 × 10^−4^	1.0 × 10^−5^	^49^
p-(AHNSA/GCE)	0–5.0 × 10^−3^	0.87 × 10^−6^	^67^
TiO2/MI-PEDOT	0–5.8 × 10^−2^	4.9 × 10^−6^	^68^
Pencil graphite electrode	7.0 × 10^−6^–1.07 × 10^−4^	2.0 × 10^−6^	^5^
CNMCP sensor	4.0 × 10^−6^–5.0 × 10^−4^	0.94 × 10^−9^	^65^
MCMCPE sensor	2.0 × 10^−6^–6.0 × 10^−4^	1.15 × 10^−7^	^31^
ACMCPE sensor	4.0 × 10^−6^–3.2 × 10^−4^	7.6 × 10^−6^	This work

### Interference study, reproducibility and stability

The interference effect on the voltammetric response for NIC was used to inspect the selectivity of the constructed sensor. It was tested in the presence of caffeine, ascorbic acid, uric acid, sucrose, lactose, and glucose, which are common in human urine and can interfere with NIC. The voltammetric response of various interferants was measured at anodic peak of Ep =  + 1050 mV, with essentially negligible current response. Due to the low concentration of other minor alkaloids (0.2–0.5 percent of total alkaloids), which cannot affect the precision of NIC detection at the sensitivity level of voltammetric tests, the interference of alkaloids that may be present in tobacco was not addressed in this work. Because cotinine and NIC are structurally related, using them together would completely define the selectivity of the proposed sensor. A constant concentration of NIC (500 μM) was spiked with similar and two-fold concentrations of the NIC biomarker "cotinine". The same experimental settings were applied for CV measurements, and recovery data ranging from 99.3% to 100% were obtained, suggesting that the modified ACMCPE might be a useful electrocatalytic sensor for detecting NIC while avoiding interference. The sensor's reproducibility was assessed by contrasting the current responses of five independent constructed ACMCPEs for 100 µM NIC. It gives a similar result, with a variation of only 4.6%. This confirms that the preparation process of the electrode is highly reliable and consistent. The long-term stability of one electrode was also tested by determining its current response every three days; the electrode was kept in the air. After two weeks, the response is 95% of its initial value, and then drops to 90% after three weeks, indicating a relatively consistent performance for NIC detection.

### Actual samples study

#### Usage of the ACMCPE sensor in urine

**Figure 8:**
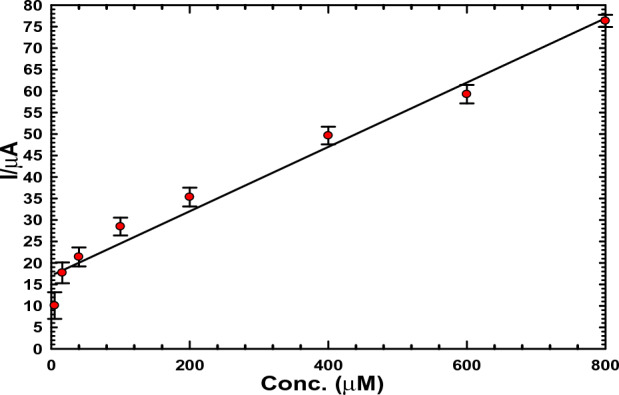
NIC calibration curve in urine.

In order to determine the methodology's applicability, the ACMCPE sensor was utilized to count the concentrations of NIC in urine samples. The calibration plot, shown in Fig. 8, produced a straight line that obeyed the linear relationship with r^2^ = 0.968, I_pa_ (µA) = 0.074 C + 17.05, and a LOD of 2.07 µM. On the calibration plot, four separate concentrations are chosen, and each is repeated five intervals to reveal the procedure's accuracy and precision. The concentration recovery was judged to be between 96.0–100.6% as shown in Table 2.

**Table 2:** The ACMCPE sensor's accuracy and precision for NIC detection in a urine sample.

(NIC) taken × 10^−6^ M	(NIC) founded^a^ (M) × 10^–6^	Recovery	RSD %
10	9.6	96.0	4.7
40	38.8	97	3.3
120	118.5	98.7	2.1
200	201.2	100.6	1.6

^a^Mean for five determinations.

#### Examination of real cigarettes samples

To guarantee that the suggested procedure was validated with actual samples, two products from different cigarette products (Marlboro and L&M) were examined for a real application. This study employed a slightly amended version of the procedure supplied by Suffredini et al.^49^. To acquire the NIC value of the tobacco sample, an appropriate amount of standard NIC solution generated in the supporting electrolyte was added to the previously formed NIC content. The recommended method's ability to quantitatively recover NIC quantities demonstrates the accuracy of the NIC detection in tobacco. The recovery data supports this assertion. (See Table 3).

**Table 3:** NIC recovery analysis in cigarette tobacco.

Cigarette brand	[NIC] taken × 10^–6^ M	[Standard] added × 10^–6^ M	Found × 10^–6^ M	Recovery (%)	RSD %
Marlboro	40	40	78.3	97.8	3.8
	80	–	119.2	99.3	1.3
	120	–	161	100.6	2.2
	160	–	201	100.5	1.7
L&M	40	40	79.2	99	2.6
	80	–	121.5	101.25	2.4
	120	–	161.2	100.75	1.8
	160	–	200.9	100.45	1.4

## Conclusions

In this study, we constructed a novel NIC sensor by electrodepositing Au-NPs onto a chitosan/CPE surface. This sensor showed outstanding NIC sensing performance due to the increase in the electro-active area developed by Au-NPs as revealed by the CV and EIS experiments. In the electrochemical NIC response, it showed a wide linear detection range (4 × 10^–6^–3.2 × 10^–4^ M) with a limit of detection 7.6 × 10^–6^ M. The sensor has a good selectivity, sensitivity, and stability for NIC determination. Interference of any accompanying substrates wasn't detected. Additionally, it has been demonstrated that the sensor can accurately identify NIC in both urine and cigarette samples. As suggestion for future, a transfer from the success of NIC detection to cotinine detection based on ACCMCPE will broaden the analytical applications as Cotinine is the metabolite of NIC and structurally similar to it. Also screen printed electrode or any other graphite derivatives as carbon nanotubes, graphene or graphene oxide for NIC detection may be applied in future investigations.

## Data Availability

The datasets used and/or analysed during the current study available from the corresponding author on reasonable request.
